# Direct Enzyme Engineering of B Family DNA Polymerases for Biotechnological Approaches

**DOI:** 10.3390/bioengineering10101150

**Published:** 2023-09-30

**Authors:** Aleksandra A. Kuznetsova, Nikita A. Kuznetsov

**Affiliations:** 1 Institute of Chemical Biology and Fundamental Medicine, Siberian Branch of Russian Academy of Sciences (SB RAS), 8 Prospekt Akad. Lavrentyeva, Novosibirsk 630090, Russia; 2 Department of Natural Sciences, Novosibirsk State University, 2 Pirogova Str., Novosibirsk 630090, Russia

**Keywords:** PCR, family B DNA polymerase, fusion DNA polymerase, mutagenesis

## Abstract

DNA-dependent DNA polymerases have been intensively studied for more than 60 years and underlie numerous biotechnological and diagnostic applications. In vitro, DNA polymerases are used for DNA manipulations, including cloning, PCR, site-directed mutagenesis, sequencing, and others. Understanding the mechanisms of action of DNA polymerases is important for the creation of new enzymes possessing improved or modified properties. This review is focused on archaeal family B DNA polymerases. These enzymes have high fidelity and thermal stability and are finding many applications in molecular biological methods. Nevertheless, the search for and construction of new DNA polymerases with altered properties is constantly underway, including enzymes for synthetic biology. This brief review describes advances in the development of family B DNA polymerases for PCR, synthesis of xeno-nucleic acids, and reverse transcription.

## 1. Introduction

DNA polymerases play a key role not only in DNA replication and repair in vivo but also in methods widely used in molecular biology, especially the polymerase chain reaction (PCR) [1,2]. PCR has revolutionized molecular biology and is now employed every day, not only in scientific research but also in the pharmaceutical industry, medicine, and diagnostics [3]. Thermostable DNA polymerases used in PCR are also widely applied in molecular biology, genetic engineering, and molecular diagnostics [4]. For genetic technologies, there is a continuous search for new enzymes that may possess improved properties compared to those known today. Mutations in DNA polymerase genes via site-directed or random mutagenesis are an effective way to construct modified enzymes with improved characteristics or specific properties for in vitro manipulations of DNA [5,6,7,8]. Another possible way to modify enzymes is to replace or add domains from other enzymes. The directed evolution of enzymes requires knowledge about the structure of a protein globule, about conserved regions, and about their functions. Techniques for polymerase engineering constantly evolve, in some cases becoming more targeted due to expanded knowledge, and in other cases, allowing for large libraries to be screened by means of more refined technologies [5,6,7,9,10,11]. This brief review deals with advancements in the development of family B DNA polymerases for PCR, synthesis of xeno-nucleic acids (XNAs), and reverse transcription.

## 2. Structure of Family B DNA Polymerases

Despite structural similarity, DNA polymerases were categorized into seven families based on phylogenetic analysis and similarity of nucleotide sequences: A, B, C, D, X, Y, and RT [12]. Family B polymerases have been found in eukaryotes, bacteria, archaea, and viruses [13,14,15,16]. Family B polymerases have been divided into many monophyletic subfamilies largely confined to a specific cellular domain [17]. Eukaryotes have four multimeric family B polymerases, namely, PolAlpha, PolDelta, PolZeta, and PolEpsilon. The best characterized archaeal family B polymerases belong to the three groups, B1, B2, and B3. In Bacteria, only one group of family B polymerases is known [17]. Based on a comprehensive analysis of family B polymerases sequences, structures, domain organizations, taxonomic distribution and co-occurrence in genomes, it was identified a new, widespread group of bacterial family B polymerases that are more closely related to the catalytically active N-terminal half of the eukaryotic PolEpsilon [17]. In Archaea, six new groups of family B polymerases were characterized. Two of them show close relationships with eukaryotic family B polymerases; the first one with PolEpsilonN, and the second one with PolAlpha, PolDelta, and PolZeta [17].

**Figure 1:**
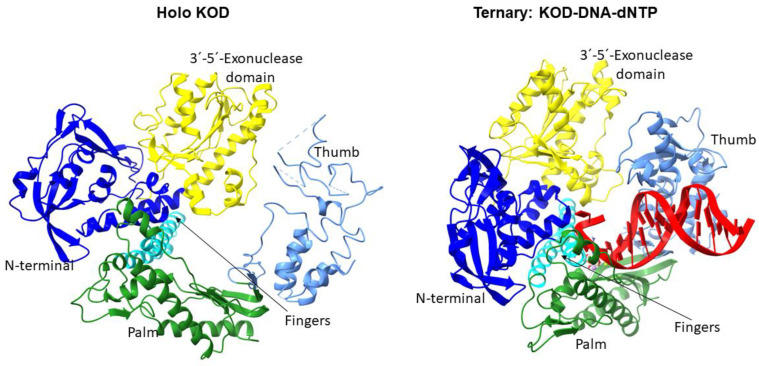
Structure of DNA polymerase KOD from *Thermococcus kodakarensis*: the *holo* enzyme (Protein Data Bank [PDB] ID: 1WNS) and a ternary closed complex (PDB ID: 5OMF). DNA is highlighted in red. Colors of DNA polymerase domains: the N-terminal domain, blue; 3′→5′-exonuclease domain, yellow; palm domain, green; fingers domain, cyan; and thumb domain, light blue.

DNA polymerases have a structure resembling a right hand. They have three main domains: the “palm”, “fingers”, and “thumb” (Figure 1). DNA binding occurs in the region at the junction of these domains. The catalytic center is based on conserved amino acid residues of the “palm” domain. The “fingers” position a template in the active site and bind dNTPs, whereas the “thumb” binds to DNA. Among all these families, the “palm” is rather conserved, whereas the “thumb” and “fingers” vary in structure [18]. 3′→5′-Exonuclease activity is manifested by a domain located independently at the N terminus of the enzyme (Figure 1). Family B polymerases do not have 5′→3′-exonuclease activity.

The first family B archaeal DNA polymerase to be characterized was *Thermococcus litoralis* Tli, well known under the trade name Vent™. To date, many archaeal DNA polymerases have been isolated and characterized [10]. There are five separate domains in the structure of family B DNA polymerases: the N-terminal domain, 3′→5′-exonuclease domain, palm domain, fingers domain, and thumb region. In Figure 1, as an example, the crystal structure of DNA polymerase KOD from *Thermococcus kodakarensis* is shown in open and closed conformations [19,20]. In the absence of DNA to replicate, DNA polymerase is in an open conformation: the fingers and thumb domains turned outwards by 33° and 24°, respectively. When DNA binds, the conformation changes from “open” to “closed”: subdomains of the thumb and fingers approach a subdomain of the palm. In the ternary complex with the correct incoming triphosphate, the enzyme is stabilized.

The 3′→5′-exonuclease domain performs a corrective function and cleaves off a terminal, erroneously incorporated nucleotide by hydrolyzing the phosphodiester bond. When a noncomplementary nucleotide is incorporated into the synthesized DNA strand, the double helix is locally unwound, thereby decreasing the stability of the complex and causing the placement of the noncomplementary nucleotide into the active site of the 3′→5′-exonuclease domain, where the phosphodiester bond is hydrolyzed. DNA binding in the active site of the 3′→5′-exonuclease domain is possible only with an open conformation of a thumb subdomain. Presumably, the mechanisms of the corrective 3′→5′-exonuclease activity and polymerase activity are coordinated by the interaction of the 3′→5′-exonuclease domain’s loop with a positively charged region of a thumb subdomain [21]. During the coordination of the 3′→5′-exonuclease domain, residue His147 is thought to interact directly with residues in the thumb subdomain. For KOD, substitution of His147 with an acidic (aspartic or glutamic) or neutrally charged amino acid residue results in enhanced binding between the 3′→5′-exonuclease domain and the edge of a thumb subdomain. In theory, His147 allows predominantly to maintain the “open” conformation required for the corrective activity.

**Figure 2:**
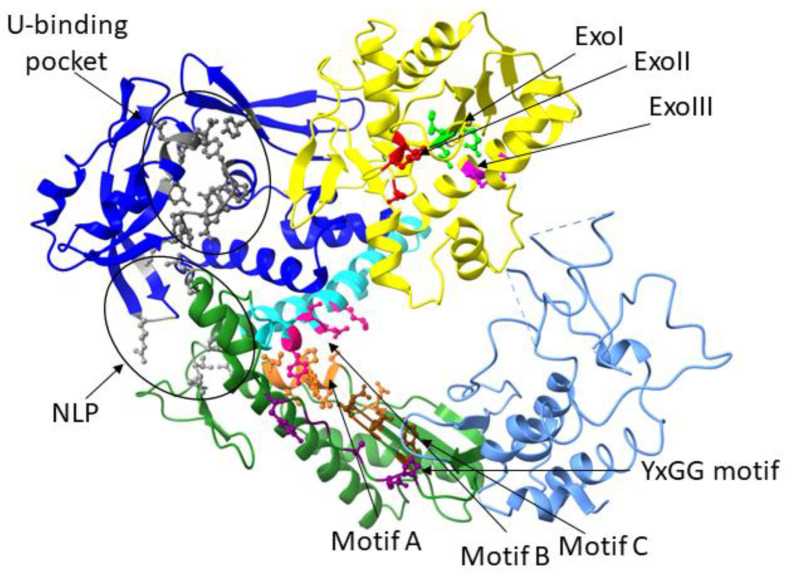
Functionally important motifs of DNA polymerase. Uracil-binding motif: grey; motifs Exo I, Exo II, and Exo III in the 3′→5′-exonuclease domain: lime, red, and magenta, respectively; polymerase motifs A, B, and C: orange, pink, and brown, respectively; motif YxGG (located between the polymerase and exonuclease domains): violet; and NPL: light grey.

The alignment of amino acid sequences has revealed the presence of highly conserved regions characteristic of family B polymerases (Figure 2). These include exonuclease motifs Exo I, II, and III in the 3′→5′-exonuclease domain; polymerase motifs A, B, and C in the palm domain; motif YxGG located between the polymerase and exonuclease domains; and the NPL motif, which arises from an interaction of amino acid residues between loops of the N-terminal domain and of the palm domain—NPL apparently participates in the corrective function [22]. It should be noted that archaeal DNA polymerases of family B are also characterized by the presence of a specialized “uracil-binding pocket” located in the N-terminal domain [23,24]. Amino acid residues forming this pocket are highly conserved and ensure specific interactions with a deaminated base. In this case, DNA replication stops, and repair systems correct the error.

Natural DNA polymerases of family B have properties that make them applicable to PCR (Table 1); however, various mutant forms and chimeric derivatives of these DNA polymerases with altered characteristics have also been described in the literature. DNA polymerases have been extensively characterized over the last decades; nevertheless, these enzymes remain partially understood. The discovering of novel chemical functions by polymerases, such as 3′-esterase activity in *Thermococcus* sp. 9°N DNA polymerase [25], is an evidence of the lack of understanding of important aspects of polymerase mechanism.

## 3. DNA Polymerases Not Blocked by 2′-Deoxyuridine

One of the specific features of family B DNA polymerases from archaea is their ability to recognize unrepaired uracil in a DNA template, and this event leads to the blockage of replication. The solution of the X-ray structure of the complex “DNA polymerase Tgo–U-containing DNA” made it possible to reveal specific features of the interaction of family B DNA polymerases with uracil-containing DNA [24,41]. During the interaction with the enzyme, uracil is everted out of the template strand and is bound in the uracil-binding pocket. At the same time, amino acid residues Tyr7 and Arg97 come into contact with the 5′- and 3′-phosphates that are directly adjacent to the uracil. The pocket has a shape that accommodates uracil and prevents the binding of standard DNA bases. The recognition of uracil is mediated by the formation of hydrogen bonds between (i) the ~N-H group of the main chain of amino acid residues Tyr37 and Ile114 and (ii) exocyclic groups O-4 and O-2 of uracil, respectively. Pro36, Pro90, and Phe116 are adjacent to the C-5 atom of uracil and prevent the stable binding of thymine through a steric effect because thymine contains a CH_3_ group at this position. Residue Val93 is in a hydrophobic α-helix (amino acid residues 90–97) that forms one side of the binding pocket. Residue Val93 directly engages in a stacking interaction with the heterocyclic ring of uracil. Although the key amino acid residues that recognize uracil are highly conserved among archaeal DNA polymerases, the mechanism by which uracil capture leads to replication termination is not fully understood. The ability of archaeal DNA polymerases to detect unrepaired uracil seems to be a safeguard against the enhanced level of cytosine deamination [23]. Contrary to nature, in PCR, the uracil-binding property is disadvantageous and can lead to a decrease in DNA amplification yields and lowered sensitivity. With the help of rational design, DNA polymerase mutant forms of the B family have been obtained that can treat uracil as normal in the matrix [23,24,42]. In this regard, the best substitutions are thought to be Pro36His, Tyr37Phe, and Val93Glu [23,42]. Mutant form Val93Glu is most commonly used in biotechnological applications because this substitution was described first. Substitution Val93Glu weakens the affinity of the enzyme for uracil-containing DNA and for dUTP in a reaction medium by more than 10-fold [23,42].

## 4. Blockage of 3′→5′-Exonuclease Activity

The balance between 3′→5′-exonuclease and polymerase activity in family B DNA polymerases is by far the best characterized. Family B DNA polymerases function while maintaining a balance between the polymerase activity and 3′→5′-exonuclease activity; this process plays a central part in the correction of a newly synthesized sequence and in the improvement of enzyme fidelity as compared to family A DNA polymerases. Mutation in highly conserved motif ExoI (Asp141Ala, Glu143Ala) blocks the 3′→5′-exonuclease activity of polymerases but does not prevent the DNA binding in the exonuclease site. When exo^−^ enzymes (3′→5′-exonuclease inactive variants) are employed in PCR, the exonuclease cleavage of primers does not take place, but a decrease in the fidelity of DNA synthesis is observed; for example, for Pfu, a reduction in fidelity by ~40-fold has been shown [26].

## 5. Increasing the Fidelity

Another active avenue of research for changing the properties of DNA polymerases is the improvement of fidelity. Minimizing errors caused by PCR is especially important for large-scale cloning projects because, with a sufficiently large pool of target DNA sequences, even high-fidelity enzymes will generate clones with mutations.

For archaeal DNA polymerases, two amino acid substitutions are known to significantly reduce the error rate. Substitution Ala408Ser results in an approximately two-fold increase in the fidelity of Pfu polymerase [43,44]. The mechanism of influence of Ala408 substitution remains unclear. This residue is located in the motif A within the dNTP-binding pocket. Residue Tyr409 of the motif A interacts with the 2′-deoxyribose residue of an incoming nucleotide, thus participating in discrimination among incoming nucleotides and blocking the binding of rNTP. For the Ala408Ser mutant form, an enhancement of affinity for dNTPs has been reported, presumably due to an additional hydrogen bond with dNTPs; in this mutant form, the researchers in question observed an improvement of fidelity by 40–50%, however, in this case, the extension rate also diminished by a factor of ~2 [43,44].

A substitution of residue His147, which is situated in the 3′→5′-exonuclease domain at the junction with the thumb, also affects the corrective activity. For DNA polymerase KOD from *Thermococcus kodakarensis*, it has been demonstrated that mutant forms containing a positively charged residue (Lys or Arg) instead of His147 have higher 3′→5′-exonuclease activity and lower polymerase activity [21]. According to the X-ray diffraction analysis performed in the same work, the presence of a constitutively positively charged residue at the 147th position reduces the stability of bonds between the 3′→5′-exonuclease domain and the thumb, thereby facilitating a transition to the open conformation, which is conducive to 3′→5′-exonuclease reactions.

## 6. Increasing Processivity

One of the most popular ways to modify an enzyme to increase its processivity is fusion with a thermostable DNA-binding protein: Sso7d from *Sulfolobus solfataricus* or Sac7d from *S. acidocaldarius* [45]. These proteins have high thermal and chemical stability and efficiently bind DNA without preference in the binding site. The fusion of Sso7d or Sac7d with DNA polymerases of families A and B increases their processivity without altering the catalytic activity and stability of the enzymes [45].

It should be noted that in the literature, researchers have described mutant forms of family B DNA polymerases possessing improved processivity that have been obtained using directed design. For instance, DNA polymerase KOD is the fastest among known archaeal polymerases of family B and is capable of elongating a DNA strand at a rate of up to 10 kbp/min [27]. The elevated processivity of the KOD enzyme may be due to the presence of seven arginines, which are believed to take part in stabilization of the binding of a primer to a template and to affect the movement of DNA between the catalytic domain and 3′→5′-exonuclease domain [46]. These arginines are clustered near the junction of the exonuclease and polymerase channels that is called a “forked point”. These seven amino acid residues are conserved among DNA polymerases of the Thermococcales order, and at two positions (243 and 264), arginine is present in all archaeal species. The remaining five arginines are more variable; it should be mentioned that Arg266 is present in both KOD and Pfu. At the remaining four positions, namely, 247, 365, 381, and 501 (where arginine is present in KOD), arginines are replaced by some other amino acid in archaeal DNA polymerases of other species [46].

In [46], an attempt was made to bring the processivity of Pfu closer to that of KOD via the introduction of additional arginines at the appropriate positions in the “forked point”. Indeed, after the introduction of additional arginines at the “branching” point, the extension rate became higher, thereby increasing processivity and improving PCR productivity. In the same work, a chimeric form of Pfu polymerase is described containing additional arginine residues and the thumb domain from KOD; this chimera also possesses enhanced processivity but still does not reach the levels of DNA polymerase KOD. In [47], for DNA polymerase Twa from *Thermococcus waiotapuensis*, analogous substitution Asn501Arg resulted in an increase of PCR efficiency, a three-fold enhancement of enzyme processivity, and a two-fold increase in the extension rate.

Several other amino acid substitutions that influence the enzymatic activity of family B DNA polymerases have been described in the literature too. For instance, for several DNA polymerases (TNA1 from *Thermococcus onnurineus* NA1, Tpa from *Thermococcus pacificus*, and Tce from *Thermococcus celericrescens*), it is reported that the replacement of residue Asn213 (which is located in the 3′→5′-exonuclease domain) with Arg raises the processivity and productivity of DNA polymerases [48,49,50]. In this context, for Tpa containing substitution Asn213Asp, a slight decrease in the fidelity of synthesis and an enhancement of affinity for dNTP were noted [50]. Nonetheless, the mechanism of influence of these substitutions remains unclear.

For DNA polymerase Pfu and some other polymerases, it has been shown that a replacement of positively charged Arg762 located at the unstructured C terminus with any neutral amino acid residue significantly improves the yield of the PCR product [44]; however, the mechanism underlying this effect is not discussed.

For DNA polymerase Twa from *Thermococcus waiotapuensis*, it has been found that substitution His633Arg leads to a two-fold increase in the processivity of the enzyme and to 1.5-fold acceleration of the extension rate, apparently owing to the stabilization of the interaction between the polymerase domain and a DNA template [51].

## 7. DNA Polymerases with Altered Substrate Specificity

DNA polymerases capable of using various modified nucleotide triphosphates as substrates are needed for various applications. It should be noted that the substrate specificity of DNA polymerases can be modulated by reaction conditions. In particular, it was shown for DNA polymerases Pab from *Pyrococcus abyssi* that replacing Mg^2+^ by Ca^2+^ lead to slower rate of phosphodiester bond formation, but the nucleotide selectivity was improved and no exonuclease degradation of the terminal nucleotides occurred [52]. The synthesis possibility of oligonucleotides containing N3′→P5′ phosphoramidate (NP) bonds (NP-DNA) was shown for DNA polymerase Bst from *B. stearothermophilus* in the presence of Ca^2+^ ions [52]. A single active site mutation Phe710Tyr enhanced the rate of NP-DNA synthesis by 21-fold.

Polymerase engineering is a powerful approach for generating polymerases with new or altered activities. For Vent DNA polymerase, the Ala488Leu mutant form has been described, which can use various dNTP derivatives as substrates [53,54]. Mutations in a similar motif of other family B DNA polymerases, including Pfu, Deep Vent, and 9°N, improve the efficiency of the incorporation of chain terminators bearing a modified part of the sugar moiety. Therminator (DNA polymerase 9°N containing the Ala485Leu mutation) has become one of the most popular enzymes for the synthesis of XNAs owing to its better ability to incorporate a variety of modified nucleoside triphosphates containing a modified base, modified sugar, or modified phosphate. Substitution Ala485Leu was originally identified as a determinant of recognition of an incoming nucleotide’s sugar moiety, but the underlying mechanism has never been determined [55]. This substitution does not significantly affect the structure of the fingers domain and does not cause any steric hindrances; however, its presence diminishes polymerase activity and fidelity as compared to the wild-type enzyme.

For DNA polymerases Tgo, KOD, Deep Vent, and 9°N, a mutant form called RI (carrying substitutions Asp141Ala, Glu143Ala, Ala485Arg, and Glu664Ile) has been described, which is capable of synthesizing TNA (from a DNA template)—this is an artificial polymer consisting of repeating α-L-threose sugars that are linked by 2′,3′-phosphodiester bonds [56]. Substitution Ala485Arg presumably promotes the rotation of the fingers domain in the direction of the DNA helix, thereby changing the geometry of the enzyme active site. Residue Glu664 contacts DNA while interacting with coordinated water molecules in the minor groove of the DNA helix. The replacement of this residue with a residue that has a hydrophobic side chain can increase the efficiency of TNA synthesis, probably owing to the weakening of contacts with the primer–template complex. A reduced ability for DNA synthesis and stronger specificity for TNA substrates was shown for KOD-RS (amino acid substitutions Asp141Ala, Glu143Ala, Ala485Arg, and Asn491Ser) and KOD-QS (amino acid substitutions Asp141Ala, Glu143Ala, Leu489Gln, and Asn491Ser) mutant forms [57]. The amino acid substitutions Ala485Arg and Asn491Ser probably allow the polymerase to adapt to the structural changes of the non-cognate TNA/DNA duplex and the incoming TNA substrate [57]. The KOD-RSGA mutant form (amino acid substitutions Asp141Ala, Glu143Ala, Ala485Arg, Asn491Ser, Arg606Gly, and Thr723Ala) demonstrated higher specificity for TNA substrates compared with KOD-RS [58,59,60].

The variants of KOD DNA polymerase (KOD DGLNK: Asn210Asp, Tyr409Gly, Ala485Leu, Asp614Asn, and Glu664Lys and KOD DLK: Asn210Asp, Ala485Leu, and Glu664Lys) were developed for improved efficiency and accuracy for LNA synthesis as well [61]. It was shown that KOD DGLNK enabled to carry out LNA synthesis from DNA (DNA → LNA), and KOD DLK enabled to carry out LNA reverse transcription to DNA (LNA → DNA). A variant of a KOD DNA polymerase E10 was found to successfully use modified substrate as 3′-O-azidomethyl-deoxyadenosine triphosphate with dye Cy3 [62]. An E10 variant was selected through semi-rational design based on the analysis of the crystal structure, catalytic mechanism, active pocket, and substrate-binding region and generating variant libraries. This mutant form includes eleven amino acid substitutions: Asp141Ala, Glu143Ala, Ser383Thr, Tyr384Phe, Val389Ile, Leu408Tyr, Ile409Ala, Ala485Glu, V589His, Thr676Lys, and Val680Met [62].

A bunch of engineered polymerase variants from *Thermococcus gorgonarius* were described that are capable of efficient synthesis of various XNAs:

- Efficient synthesis of tPhoNA (3′-2′-phosphonomethyl-threosyl nucleic acid) was described for the TgoT EPFLH, which contains mutations Val93Gln, Asp141Ala, Glu143Ala, His147Glu, Leu403Pro, Leu408Phe, Ala485Leu, Ile521Leu, and Glu664His [63].
- Another variant of DNA polymerase Tgo is the TGLLK mutant form, which contains substitutions Val93Gln, Asp141Ala, Glu143Ala, Ala485Leu, Tyr409Gly, Ile521Leu, Phe545Leu, and Glu664Lys and can perform template-dependent DNA and RNA synthesis with regioisomeric 2′-5′-bonds [64].
- Efficient synthesis of XNAs—in which the canonical ribofuranose ring is substituted with five- or six-membered congeners constituting 1,5-anhydrohexitol nucleic acids—was documented for the Pol6G12 mutant form, which contains 18 amino acid substitutions (Val93Gln, Asp141Ala, Glu143Ala, Ala485Leu, Val589Ala, Glu609Lys, Ile610Met, Lys659Gln, Glu664Gln, Gln665Pro, Arg668Lys, Asp669Gln, Lys671His, Lys674Arg, Thr676Arg, Ala681Ser, Leu704Pro, and Glu730Gly) [65].
- Efficient synthesis of CeNAs (cyclohexenyl nucleic acids) and of LNAs (2′-O,4′-C-methylene-b-D-ribonucleic acids, i.e., locked nucleic acids) was described for the PolC7 mutant form (amino acid substitutions Val93Gln, Asp141Ala, Glu143Ala, Ala485Leu, Glu654Gln, Glu658Gln, Lys659Gln, Val661Ala, Glu664Gln, Gln665Pro, Asp669Ala, Lys671Gln, Thr676Lys, and Arg709Lys) [65].
- Efficient synthesis of ANAs (arabinonucleic acids) and of FANAs (2′-fluoro-arabinonucleic acids) has been described for the PolD4K mutant form (amino acid substitutions Val93Gln, Asp141Ala, Glu143Ala, Ala485Leu, Leu403Pro, Pro657Thr, Glu658Gln, Lys659His, Tyr663His, Glu664Lys, Asp669Ala, Lys671Asn, and Thr676Ile) [65].
- Efficient DNA-templated synthesis of uncharged P-methyl and P-ethyl phosphonate nucleic acids (phNAs) has been described for the RT521 mutant form (amino acid substitutions Val93Gln, Asp141Ala, Glu143Ala, Ala485Leu, Glu429Gly, Ile521Leu, and Lys726Arg) and GV2 mutant form (amino acid substitutions Val93Gln, Asp141Ala, Glu143Ala, Ala485Leu, Glu429Gly, Ile521Leu, and Lys726Arg, Lys487Gly, Arg606Val, and Arg613Val) [66].
- Efficient synthesis of 2′-MOE-RNA and 2′-OMe-RNA has been described for the 2M mutant form (amino acid substitutions Val93Gln, Asp141Ala, Glu143Ala, Ala485Leu, Tyr409Gly, Ile521Leu, Phe545Leu, Glu664Lys, Thr541Gly, and Lys592Ala) and 3M mutant form (amino acid substitutions Val93Gln, Asp141Ala, Glu143Ala, Ala485Leu, Tyr409Gly, Ile521Leu, Phe545Leu, Glu664Lys, Thr541Gly, Lys592Ala, and Lys664Arg) [67].

Besides, Tgo mutant forms capable of performing the reverse synthesis of DNA on an XNA strand were described: RT521 mutant form (amino acid substitutions Val93Gln, Asp141Ala, Glu143Ala, Ala485Leu, Glu429Gly, Ile521Leu, and Lys726Arg) and RT521K mutant form (amino acid substitutions Val93Gln, Asp141Ala, Glu143Ala, Ala485Leu, Glu429Gly, Ile521Leu, Lys726Arg, Ala385Val, Phe445Leu, and Glu664Lys) [65]. It must be pointed out that amino acid substitutions that ensure the synthesis of XNA in the DNA template are clustered at the periphery of the region of interaction with a primer–template duplex in the thumb domain at a distance of >20 Å from the enzyme active site. Those authors noted the importance of amino acid substitution Ile521Leu for the function of reverse transcription from XNA into DNA. The overall fidelity (defined as error rate per position) of a complete DNA → XNA → DNA replication cycle ranged from 4.3 × 10^−3^ to 5.3 × 10^−2^ [65]. The reverse synthesis of DNA on a tPhoNA strand was performed with RT521 mutant form and with KOD variant, namely K.RT521K mutant form (amino acid substitutions Val93Glu, Asp141Ala, Glu143Ala, Ala485Leu, Ile521Leu, and Glu664Lys), with an error rate of 17–20 × 10^−3^ [63].

The general strategy for the directed evolution of RT function for any template chemistry called compartmentalized bead labelling is described in [68]. The directed evolution of RT521K allowed to obtain an efficient RTs for 2′-O-methyl RNA, for hexitol nucleic acids, orphan XNA chemistries D-altritol nucleic acid and 2′-methoxyethyl RNA: RT-TKK mutant form (RT521K + Ile114Thr, Ser383Lys, and Asn735Lys), RT-C8 mutant form (RT521K + Ile114Thr, Ser383Lys, Asn735Lys, Phe493Val, Tyr496Asn, Tyr497Leu, Tyr499Ala, Ala500Gln, and Lys501His) and its RTC8-exo^+^ variant, RT-H4 mutant form (RT521K + Ile114Thr, Ser383Lys, Asn735Lys, Phe493Val, Tyr496His, Tyr497Met, Tyr499Phe, Ala500Glu, and Lys501Asn) and its RTH4-exo^+^ variant, RT-TR mutant form (RT521K + Pro410Thr and Ser411Arg) [68].

Table 2 summarizes the amino acid substitutions of engineered DNA polymerases with altered substrate specificity. Other recent reviews are available that provide a broad overview of different aspects and structural analysis of engineered DNA polymerases for XNA synthesis [5,6,9,69]. An initial attempt to compare the substrate specificity, thermal stability, reverse transcriptase activity, and fidelity of laboratory-evolved polymerases that were established to synthesize RNA and five different examples of XNA polymers was performed in [59]. This particular work compared the enzymes in conditions optimal for TNA synthesis by the engineered polymerases. All together, these data benchmark the activity of different DNA polymerases and provide opportunities for generating new polymerase variants.

### 7.1. DNA Polymerases with Elevated Sensitivity to Methylated DNA

For DNA polymerase KOD-exo^−^, mutant forms with a substitution of Gly245 have been described that can selectively distinguish between C and 5-methyl-cytosine (5mC) in a DNA template [72]. The mechanism by which this amino acid substitution alters the efficiency of incorporation of dGTP opposite to C and 5mC remains unclear. The amino acid residue Gly245 is located in the 3′→5′ loop of the exonuclease domain near the 5′ end of the template strand. Those authors hypothesized that a substitution of glycine at this position with another amino acid residue increases the likelihood of additional interactions (van der Waals or polar) with the substrate as compared to the wild-type enzyme. According to their calculations, after the substitution of Gly245Asp, the formation of hydrogen bonds with the nitrogenous base at position +2 of the template strand becomes possible, thereby causing conformational changes, altering the orientation of the template in the active site and thus, possibly affecting the differences in the incorporation of nucleotides opposite C and 5mC [72].

In [73], other mutant forms of KOD-exo^−^, namely, Arg501Cys, Arg606Gln, and Arg606Trp, are described that show increased sensitivity to the presence of mismatches and can discriminate between C and 5 mC. The substitution of Arg606 led to an increase in the number of selective contacts with a primer, whereas the substitution of Arg501 resulted in an increase in the number of selective contacts with the template owing to the removal of positive charges, which probably stabilize the interactions of the enzyme with the primer–template duplex, regardless of whether it contains a mismatch. The loss of potential electrostatic interactions and a less polar environment enhance the selectivity of the enzyme and its sensitivity to the presence of a mismatch [73].

### 7.2. DNA Polymerases with Properties of RNA Polymerase

For DNA polymerase 9°N (Therminator pol) from *Thermococcus species*, mutant form Leu408Gln has been described, which can perform RNA synthesis on a DNA template [71]. Amino acid residue Leu408 is located in motif A of the polymerase domain and apparently participates in substrate discrimination. Tyr409, which is located nearby in the wild-type enzyme, prevents ribonucleotide incorporation through a steric conflict with the 2′-OH group of incoming rNTP. By contrast, for the Leu408Gln mutant form, there appears to be no steric conflict with the 2′-OH group of the incoming ribonucleotide, resulting in stronger affinity for rNTP and the possibility of RNA strand synthesis. Moreover, mutant form Leu408Gln can use C5-modified deoxyribonucleoside triphosphates as substrates in DNA synthesis [71].

For DNA polymerases Tgo, KOD, Deep Vent, and 9°N, mutant form QGLK has been described, which can efficiently synthesize RNA on a DNA strand. QGLK mutant forms of these enzymes carry the following substitutions (amino acid numbering is given for the Tgo enzyme): Asp141Ala, Glu143Ala, Val93Gln, Tyr409Gly, Ala485Leu, and Glu664Lys. In this context, substitutions Asp141Ala and Glu143Ala, as in the previous cases, are necessary to block 3′→5′-exonuclease activity; substitution Val93Gln is necessary to prevent the blockage of the enzyme by uracil-containing DNA, and the remaining substitutions are required for the emergence of RNA polymerase activity [56,70]. The fidelity of QGLK polymerases was found to be 1–5 incorrect nucleotides per 1000 incorporation events [56].

### 7.3. DNA Polymerases with Reverse-Transcriptase Properties

An interesting example of directed enzyme engineering is the construction of a reverse transcriptase based on DNA polymerase KOD [74,75]. Directed evolution combined with an analysis of structural data and modeling revealed residues that may be responsible for the enzyme’s ability to perform reverse-transcriptase functions. The desired enzyme, which was named reverse transcription xenopolymerase (RTX), contains 16 amino acid substitutions: Phe38Leu, Arg97Met, Lys118Ile, Met137Leu, Arg381His, Tyr384His, Val389Ile, Lys466Arg, Tyr493Leu, Thr514Ile, Ile521Leu, Phe587Leu, Glu664Lys, Gly711Val, Asn735Lys, and Trp768Arg. The resulting RTX has RNA- and DNA-dependent DNA polymerase activities. The binding of an RNA–DNA hybrid proved to be less efficient than the binding of a DNA duplex; the synthesis on an RNA strand was also less efficient than the synthesis on a DNA template. It should be noted that the enzyme retained thermal stability and the corrective 3′→5′-exonuclease activity. The fidelity of polymerase RTX is ~3.5 × 10^−5^ [74]. Structural analysis of RTX in complex with either a DNA duplex or an RNA–DNA hybrid and a comparison with structures of the original KOD in the form of the *apo*-enzyme or a binary or ternary complex helped to advance several hypotheses about the functional significance of the analyzed amino acid substitutions [75]. It was revealed that among the 16 substitutions that gave rise to the reverse-transcriptase activity, only six are in the substrate-binding region, and the others change domain–domain interactions in protein structure of the enzyme; as a consequence, the binding of an RNA–DNA hybrid and a reverse-transcription reaction become possible. Those authors theorized [75] that the flexibility of the thumb domain plays a key role in the emergence of the reverse-transcriptase function.

## 8. Conclusions

Polymerases underlie many biotechnological and medical applications, from PCR assays to selection of aptamers and from DNA sequencing to *de novo* DNA synthesis. Archaeal family B DNA polymerases have high fidelity and thermal stability and are powerful molecular biological tools with a wide range of capabilities. At present, archaeal DNA polymerases are widely used for in vitro DNA manipulations, including DNA amplification, sequencing, site-directed mutagenesis, and other applications. Our analysis of literature data indicates that the design by protein engineering methods and the creation of mutant and/or chimeric forms of DNA polymerases possessing improved characteristics is a hot area in the field of modern biology. Nonetheless, the discovery and characterization of new DNA polymerases is an important area of research too.

Knowledge of the structure of DNA polymerases is important for the directed engineering of enzymes. Complexes of DNA polymerases with modified substrates and nucleoside triphosphate analogs can provide information about the steric restrictions that can affect functions of the enzyme. Detailed insights into the mechanism of action of DNA polymerases have a direct impact on the development of new enzymes with modified properties. The current engineered DNA polymerases already possess improved or artificial activities often not present in the natural DNA polymerases. The functional requirements underlying the engineering of new enzymes have already led to the creation of incredible molecular machines that can be improved further. More efforts are needed to develop improved polymerases and discover their application as useful tools in new application areas.

## Figures and Tables

**Table 1:** Characteristics of thermostable DNA polymerases of family B.

pol	Archaea	Optimal Temperature (°C)	Extension Rate (kbp/min)	Error Rate (×10^−6^)	Thermal Stability (τ_1/2_)	Ref.
Deep Vent™	*Pyrococcus* species GB-D	72–75	1.4	12–2.7 [exo^−^: 200]	95 °C/23 h; 100 °C/8 h	[26]
KOD	*Thermococcus kodakarensis*	72–75	6.0–7.8	2.6	95 °C/12 h; 100 °C/3 h	[27]
Pab (Isis™)	*Pyrococcus abyssi*	70–80	n.p.	0.7–6.7	100 °C/5 h	[28]
Pfu	*Pyrococcus furiosus*	72–80	0.5–1.5	0.7–2.2 [exo^−^: 20–60]	95 °C/95% after 1 h	[26]
Pwo	*Pyrococcus woesei*	72	n.p.	n.p.	100 °C/2 h	[29]
Tfu	*Thermococcus fumicolans*	72	0.32	9–53	95 °C/3.3 h 100 °C/2 h	[30]
Tgo	*Thermococcus gorgonarius*	72	1.5	3.5–5.6	95 °C/2 h	[31]
Tli (Vent™)	*Thermococcus litoralis*	72–80	1.0	2.8–45 [exo^−^: 190]	95 °C/6.7 h 100 °C/1.8 h	[26,32]
Tma (UlTma™)	*Thermotoga maritima*	65–75	n.p.	32–74	97.5 °C/50 min	[33]
TNA1	*Thermococcus* sp. NA1	75	3.6	220	95 °C/12.5 h 100 °C/3.5 h	[34]
Tne	*Thermotoga neopolitana*	72–75	n.p.	n.p.	90 °C/1 h	[35]
Tpe	*Thermococcus peptonophilus*	75	2.0	3.4	95 °C/4 h	[36]
Tzi (Pfx50™)	*Thermococcus zilligii*	68	0.5–1.0	2.0	n.p.	[37]
Tga	*Thermococcus gammatolerans*	45–65	n.p.	n.p.	95 °C/93% after 1 h; 20% after 1 h	[38]
Pca	*Pyrobaculum* *calidifontis*	75	n.p.	n.p.	95 °C/4.5 h 100 °C/0.5 h	[39]
Iho	*Ignicoccus hospitalis* KIN4/I	70	n.p.	14.2	94 °C/2 h	[40]

n.p.: no published data.

**Table 2:** Summary of engineered DNA polymerases.

DNA Polymerase	Amino Acid Substitutions	Properties	Ref.
Pfu, Tgo, Tli	Pro36His, Tyr37Phe, Val93Glu	Treating uracil as normal in the matrix	[23,24,42]
Pfu, KOD	Ala408Ser, Hys147Lys/Arg	Increase in the polymerase fidelity	[21,43,44]
Pfu, Twa, TNA1, Tpa, Tce	Leu381Arg, Asn502Arg Asn213Arg/Asp His633Arg	An increase in PCR efficiency, enhancement of enzyme processivity, increase in the extension rate.	[46,47,48,49,50,51]
Vent, Pfu, Deep Vent, 9°N	Ala488Leu	Increase incorporation of modified dNTP containing a modified base, modified sugar, or modified phosphate.	[53,54,55]
Tgo, KOD, Deep Vent, 9°N	**RI**: Asp141Ala, Glu143Ala, Ala485Arg, Glu664Ile	Synthesis TNA from a DNA template	[56]
	**QGLK**: Asp141Ala, Glu143Ala, Val93Gln, Tyr409Gly, Ala485Leu, Glu664Lys	Efficiently synthesis RNA on a DNA strand	[56,70]
9°N	Leu408Gln	RNA synthesis on a DNA template Increase incorporation of C5-modified dNTP	[71]
Tgo	**EPFLH**: Val93Gln, Asp141Ala, Glu143Ala, His147Glu, Leu403Pro, Leu408Phe, Ala485Leu, Ile521Leu, Glu664His	Synthesis tPhoNA on a DNA strand and DNA on a tPhoNA strand	[63]
	**TGLLK**: Val93Gln, Asp141Ala, Glu143Ala, Ala485Leu, Tyr409Gly, Ile521Leu, Phe545Leu, Glu664Lys	Template-dependent DNA and RNA synthesis with regioisomeric 2′-5′-bonds	[64]
	**Pol6G12**: Val93Gln, Asp141Ala, Glu143Ala, Ala485Leu, Val589Ala, Glu609Lys, Ile610Met, Lys659Gln, Glu664Gln, Gln665Pro, Arg668Lys, Asp669Gln, Lys671His, Lys674Arg, Thr676Arg, Ala681Ser, Leu704Pro, Glu730Gly	Efficient synthesis of XNAs	[65]
	**PolC7**: Val93Gln, Asp141Ala, Glu143Ala, Ala485Leu, Glu654Gln, Glu658Gln, Lys659Gln, Val661Ala, Glu664Gln, Gln665Pro, Asp669Ala, Lys671Gln, Thr676Lys, Arg709Lys	Efficient synthesis of CeNAs and LNAs	[65]
	**PolD4K**: Val93Gln, Asp141Ala, Glu143Ala, Ala485Leu, Leu403Pro, Pro657Thr, Glu658Gln, Lys659His, Tyr663His, Glu664Lys, Asp669Ala, Lys671Asn, Thr676Ile	Efficient synthesis of ANAs and FANAs	[65]
	**GV2**: Val93Gln, Asp141Ala, Glu143Ala, Ala485Leu, Glu429Gly, Ile521Leu, Lys726Arg, Lys487Gly, Arg606Val, Arg613Val	Efficient synthesis of phNAs	[66]
	**2M:** Val93Gln, Asp141Ala, Glu143Ala, Ala485Leu, Tyr409Gly, Ile521Leu, Phe545Leu, Glu664Lys, Thr541Gly, Lys592Ala	Efficient synthesis of 2′-MOE-RNA, 2′-OMe-RNA	[67]
	**3M:** Val93Gln, Asp141Ala, Glu143Ala, Ala485Leu, Tyr409Gly, Ile521Leu, Phe545Leu, Glu664Lys, Thr541Gly, Lys592Ala, Lys664Arg	Efficient synthesis of 2′-MOE-RNA, 2′-OMe-RNA	[67]
	**RT521**: Val93Gln, Asp141Ala, Glu143Ala, Ala485Leu, Glu429Gly, Ile521Leu, Lys726Arg	Efficient synthesis of XNAs Reverse synthesis of DNA on an XNA strand	[63,65,66]
	**RT521K**: Val93Gln, Asp141Ala, Glu143Ala, Ala485Leu, Glu429Gly, Ile521Leu, Lys726Arg, Ala385Val, Phe445Leu, Glu664Lys	Reverse synthesis of DNA on an XNA strand	[65]
	**RT-TKK:** Val93Gln, Asp141Ala, Glu143Ala, Ala485Leu, Glu429Gly, Ile521Leu, Lys726Arg, Ala385Val, Phe445Leu, Glu664Lys, Ile114Thr, Ser383Lys, Asn735Lys	Reverse synthesis of DNA on a 2′-OMe-RNA or AtNA strand	[68]
	**RT-C8:** Val93Gln, Asp141Ala, Glu143Ala, Ala485Leu, Glu429Gly, Ile521Leu, Lys726Arg, Ala385Val, Phe445Leu, Glu664Lys, Ile114Thr, Ser383Lys, Asn735Lys, Phe493Val, Tyr496Asn, Tyr497Leu, Tyr499Ala, Ala500Gln, Lys501His	Reverse synthesis of DNA on 2′-OMe-RNA, HNA, AtNA, 2′-MOE-RNA or PS 2′-MOE-RNA strand	[68]
	**RTC8-exo^+^**: Val93Gln, Ala485Leu, Glu429Gly, Ile521Leu, Lys726Arg, Ala385Val, Phe445Leu, Glu664Lys, Ile114Thr, Ser383Lys, Asn735Lys, Phe493Val, Tyr496Asn, Tyr497Leu, Tyr499Ala, Ala500Gln, Lys501His	Reverse synthesis of DNA on 2′-OMe-RNA, HNA, AtNA, 2′-MOE-RNA or PS 2′-MOE-RNA strand	[68]
	**RT-H4**: Val93Gln, Asp141Ala, Glu143Ala, Ala485Leu, Glu429Gly, Ile521Leu, Lys726Arg, Ala385Val, Phe445Leu, Glu664Lys, Ile114Thr, Ser383Lys, Asn735Lys, Phe493Val, Tyr496His, Tyr497Met, Tyr499Phe, Ala500Glu, Lys501Asn	Reverse synthesis of DNA on HNA strand	[68]
	**RTH4-exo^+^**: Val93Gln, Ala485Leu, Glu429Gly, Ile521Leu, Lys726Arg, Ala385Val, Phe445Leu, Glu664Lys, Ile114Thr, Ser383Lys, Asn735Lys, Phe493Val, Tyr496His, Tyr497Met, Tyr499Phe, Ala500Glu, Lys501Asn	Reverse synthesis of DNA on HNA strand	[68]
	**RT-TR**: Val93Gln, Asp141Ala, Glu143Ala, Ala485Leu, Glu429Gly, Ile521Leu, Lys726Arg, Ala385Val, Phe445Leu, Glu664Lys, Pro410Thr, Ser411Arg	Reverse synthesis of DNA on 2′-OMe-RNA, HNA, AtNAs, 2′-MOE-RNA or PS 2′-MOE-RNA strand with enhanced fidelity	[68]
KOD	**DGLNK**: Asn210Asp, Tyr409Gly, Ala485Leu, Asp614Asn, Glu664Lys	Efficient synthesis of LNA	[61]
	**DLK**: Asn210Asp, Ala485Leu, Glu664Lys	Efficient synthesis of LNA Reverse synthesis of DNA on LNA strand	[61]
	**RS:** Asp141Ala, Glu143Ala, Ala485Arg, Asn491Ser	Efficient synthesis of TNA	[57]
	**QS:** Asp141Ala, Glu143Ala, Leu489Gln, Asn491Ser	Efficient synthesis of TNA	[57]
	**RSGA:** Asp141Ala, Glu143Ala, Ala485Arg, Asn491Ser, Arg606Gly, Thr723Ala	Efficient synthesis of FANA, ANA, HNA, TNA, C5-modified TNA, and PMT	[58,59,60]
	**E10**: Asp141Ala, Glu143Ala, Ser383Thr, Tyr384Phe, Val389Ile, Leu408Tyr, Ile409Ala, Ala485Glu, V589His, Thr676Lys, Val680Met	Efficient incorporation of modified dNTP	[62]
	Exo^–^ variant + Gly245Asp	5-methyl-cytosine sensitive	[72]
	Exo^–^ variant + Arg501Cys, Arg606Gln/Trp	Mismatches/5-methyl-cytosine sensitive	[73]
	**K.RT521K**: Val93Glu, Asp141Ala, Glu143Ala, Ala485Leu, Ile521Leu, Glu664Lys	Reverse synthesis of DNA on tPhoNA strand	[63]
	**RTX**: Phe38Leu, Arg97Met, Lys118Ile, Met137Leu, Arg381His, Tyr384His, Val389Ile, Lys466Arg, Tyr493Leu, Thr514Ile, Ile521Leu, Phe587Leu, Glu664Lys, Gly711Val, Asn735Lys, Trp768Arg.	RNA- and DNA-dependent DNA polymerase activities	[74,75]
